# 20 years of alveolar distraction: A systematic review of the literature

**DOI:** 10.4317/medoral.22645

**Published:** 2018-11-21

**Authors:** Mario Pérez-Sayáns, José-Manuel Martínez-Martín, Cintia Chamorro-Petronacci, Mercedes Gallas-Torreira, Xabier Marichalar-Mendía, Abel García-García

**Affiliations:** 1 DDS, PhD. Oral Medicine, Oral Surgery and Implantology Unit. Faculty of Medicine and Dentistry. Instituto de Investigación Sanitaria de Santiago (IDIS), Santiago de Compostela, Spain. Address: Entrerríos s/n, Santiago de Compostela C.P. 15782 Spain; 2 DDS, PhD. Master de Medicina Oral, Cirugía oral e Implantología, University of Santiago de Compostela. Santiago de Compostela, Spain; 3 DDS, PhD. Oral Medicine, Oral Surgery and Implantology Unit. Faculty of Medicine and Dentistry. Instituto de Investigación Sanitaria de Santiago (IDIS), Santiago de Compostela, Spain; 4 PhD. Department of Stomatology II. Faculty of Medicine and Odontology. Barrio Sarriena s/n. 48940 Leioa, Bizkaia. Spain; 5 MD, PhD. Professor of Maxillofacial Surgery, Oral medicine, oral surgery and implantology, University of Santiago de Compostela. Department of Maxillofacial Surgery, Complejo Hospitalario Universitario de Santiago de Compostela, Spain

## Abstract

**Background:**

The Vertical Alveolar Distraction Osteogenesis (VADO) technique is an excellent solution for bone and soft tissue neoformation in areas in which there has been significant bone atrophy that hinders normal rehabilitation using dental implants. The goal of this systematic review is to analyze the most relevant articles published on VADO in the literature over the past 20 years.

**Material and Methods:**

The review was performed by using the keywords: “alveolar ridge”, “distraction ostegenesis” and “dental implant”. This search produced a total result of 240 articles. The clinical studies and cases reported in humans amounted to 113 articles, 18 articles referred to studies developed on animals and 33 review articles. The presentation of this systematic analysis follows the criteria described in the PRISMA declaration.

**Results:**

22 articles complied with the inclusion criteria and 7 articles more were added manually, reaching a total sample of 29 studies. Following the analysis of the studies, they were classified into 18 high-quality, 10 mediumquality and 1 low-quality study. Only 4 studies achieved a maximum score of 9 (according to NewCastle Ottawa Scale, NOS).

**Conclusions:**

VADO is a technique with greater potential in vertical gain. The performance of dental implants has a success and survival rate similar to dental implants placed on bones that are not subject to increase techniques.

** Key words:**Vertical distraction osteogenesis, alveolar ridge, distraction osteogenesis, dental implant.

## Introduction

The Alveolar Distraction Osteogenesis (ADO) technique is an excellent solution for bone and soft tissue neoformation in areas in which there has been significant bone atrophy that hinders normal rehabilitation using dental (1). After performing an osteotomy in the alveolar bone, a distraction device is fixed on the transport segment and the basal bone. The transport segment remains vascularized thanks to the blood flow from the periosteum, which must be preserved on the side of the tongue. Subsequently, the transport segment is submitted to gradual traction to separate it from the basal bone. This traction activates the phenomena that will ultimately lead to the regeneration of tissue following the progressive maturity of the distraction callus created during this process. The resulting bone mass and shape shall depend on the distraction vector, the mechanical forces and the blood flow.

Several articles have specifically studied the biology of osteogenesis (2). Chiapasco *et al.* reported that after 12 weeks of consolidation, the percentage of mineralized bone in the distraction gap ranged from 21.6% to 57.8%. The newly formed bone was placed perpendicular to the osteotomy line and consisted of a non-laminar reinforced bone of parallel collagen fibers (3). Tuker *et al.* obtained similar histological results after 12 weeks of consolidation. They correlated the histological results with panoramic X-Rays (OPG) and computerized tomography (CT), to develop an analysis of bone density. Once the distraction had finished, the OPG showed a radiolucid area in the distraction gaps. After 12 weeks of consolidation, these distraction gaps showed some radio-opaque areas with radio-lucid areas, after a year, their appearance was similar to pre-existing bones. CT-scans showed that new bones after 12 weeks of consolidation were denser than medullary bones (4). Amir *et al.* found a correlation between the volume of the blood vessels and the bone density of the newly formed bone, concluding that an appropriate blood flow is essential for the development, remodeling and regeneration of the bone (5).

Following the initial enthusiasm, ADO procedures have been gradually abandoned by most clinicians due to the discomfort of their patients, the numerous complications and the need for the patient to comply strictly with the protocol (6).

ADO has been applied for 20 years as a bone regeneration technique. In general, we can distinguish 2 types of distraction devices: intraosseous and extraosseous. They can also be differentiated depending on their role, dividing them into distractors or distractor-implants (7). Depending on the direction of the regenerated bone, they are divided into vertical or horizontal distraction devices. Different studies present different distraction protocols for each distractor device.

Esposito *et al.*, in a systematic review of Cochrane on different vertical regeneration techniques, didn’t find sufficient evidence regarding which was the best procedure. However, they reported that the ADO technique has the greatest potential for vertical regeneration procedures (7).

The goal of this systematic review is to analyze the most relevant articles published on Vertical Alveolar Distraction Osteogenesis in the literature over the past 20 years. We have focused on location of the distraction device, cause for the ADO, distraction sequence, vertical gain obtained by the end of the procedure, number of implants placed, follow-up period, implant success and survival.

## Material and Methods

Information sources: A systemic review was performed using MEDLINE data bases (via PubMed, from January 1996 to November 2016) and the Cochrane Library (CL) (from January 1996 to November 2016). This search was performed during December 2016.

Search strategy The search in PubMed via Medline was based on the following terms (alveolar ridge OR distraction osteogenesis AND dental implant). The search used both MeSH terms (Medical Subject Headings) and free search. The presentation of this systematic analysis follows the criteria described in the PRISMA (Preferred Reporting Item for Systematic Review and Meta-Analyses) declaration (8).

PICO methodology: adults (P=patients); treated (I=intervention), with different type of distractors (C=comparison), associated with vertical gain (O=result). PICO question: In patients treated with ADO, is there differences in vertical gain according to the distractor used?

Process for selecting studies: A group of research experts in oral (MP-S and JM-M) and maxillofacial surgeon (AG-G) have selected studies according to the following criteria: 1) contained more than 7 patients; 2) detailed the distraction device used. Consensus between the main researchers was acceptable during the inclusion process and the agreement in this process was calculated with Cohen’s Kappa coefficient, obtaining a k score of 0.8.

Method of data extraction: To value the quality of the studies, the following data were extracted and analyzed: indication of the location of the distractor, indication of the cause for the ADO, specification of the used distraction sequence (latency, activation and consolidation period), Indication of the vertical gain obtained by the end of the distraction, description of the complications observed during the procedure, number of implants placed, follow-up period of 12 months, implant success, implant survival. Quality was assessed using the Newcastle-Ottawa Scale (NOS) with a total of nine items. In the analysis the studies were defined as medium quality: 4-6, low quality: 1-3, high quality 7-9.

Summary measures: To classify complications deriving from the ADO technique, we have used the proposed by Enislidis *et al.* (6), dividing the complications between major and minor, this way we achieved a better quantification.

## Results

The search produced a total result of 240 articles. The clinical studies and cases reported in humans amounted to 113 articles, 18 articles referred to studies developed on animals and 33 review articles.

A total of 29 studies were included in our review, 22 articles complied with the inclusion criteria and 7 articles more were added manually (4,9-14). On the basis of this review, 18 articles were classified as high-quality, 10 as medium-quality and 1 as low-quality study. Only 4 studies achieved a maximum score of 9 (15,17,30,18) ( Table 1).

**Table 1:**
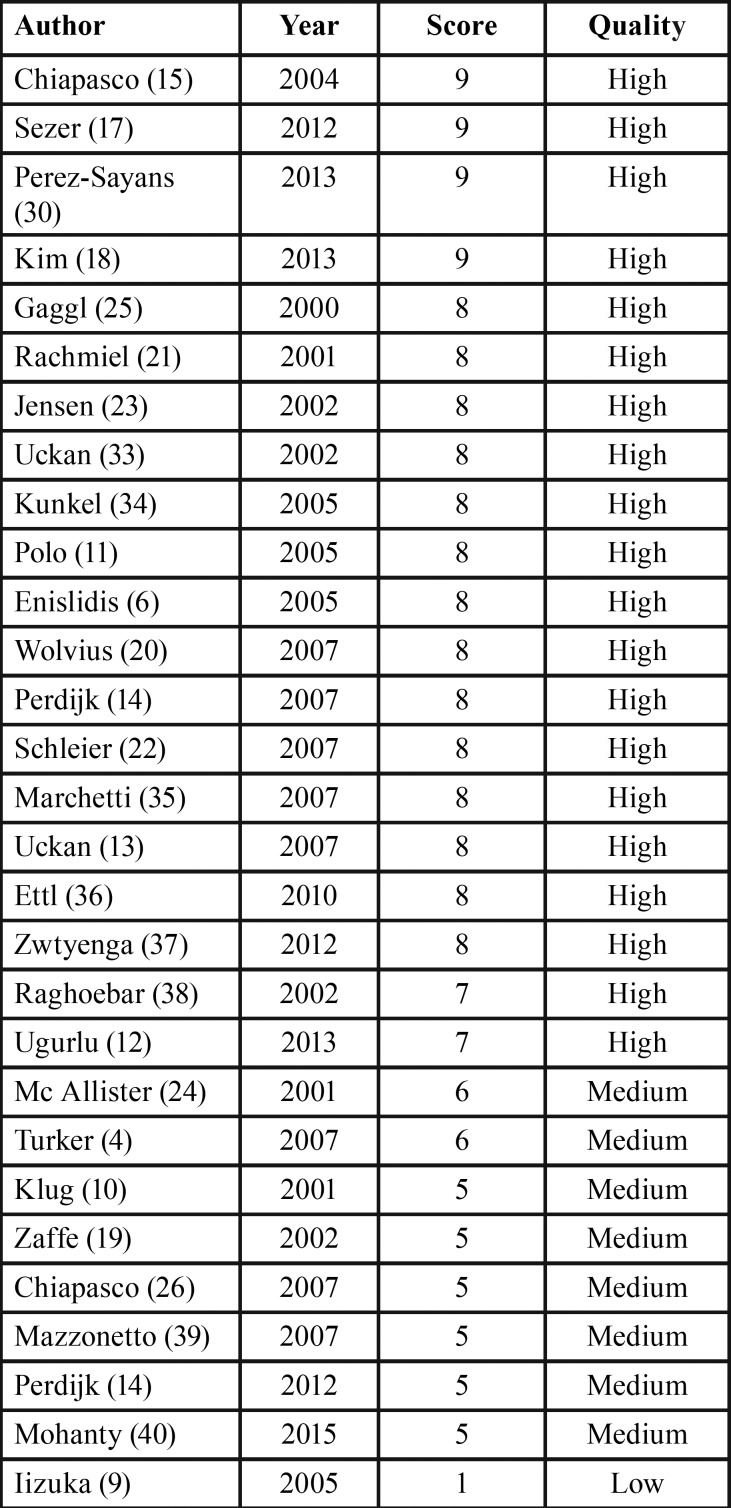
Quality score.

-Devices

In the review we found 12 different types of distraction devices:

_7 intraosseous distractors: DISIS (SIS Trade, Systems, Klagenfurt, Austria), OGD (ACE Surgical Supply, Brockton, MA), LEAD (Leibinger Endosseous Alveolar Distraction System, Freiburg, Germany), OSTEOMED (Osteomed Quick-fix System, Osteomed, Dallas, TX), 3i (3i Implant-distractor, Implant Innovations, West Palm Beach, FL), GDD (GDD, Martin Medizin Technik, Tuttlingen, Germany), MAINZ (‘Mainz’-Distractor; Medicon eG, Tuttlingen, Germany).

_5 extraosseous distractors: TRACK (Track® Distractor 1.0 or 1.5 mm, Martin, Tuttlingen, Germany), V2 (V2-Alveolar Distraction System, Medartis, AG, Basel, Switzerland), MONDEAL (Mondeal Medical Systems GmbH, Tuttlingen, Germany), MODUS (Modus; Medartis AG, Basel, Switzerland), CONEXAO (Conexao1, Implant System, Sao Paulo, Brazil), 

Among the 29 studies included in this review, a total of 618 patients were treated; 424 of them had been treated with an extraosseous distractor and 194 with an intraosseous distractor ( Table 2,  Table 2 continue).

**Table 2:**
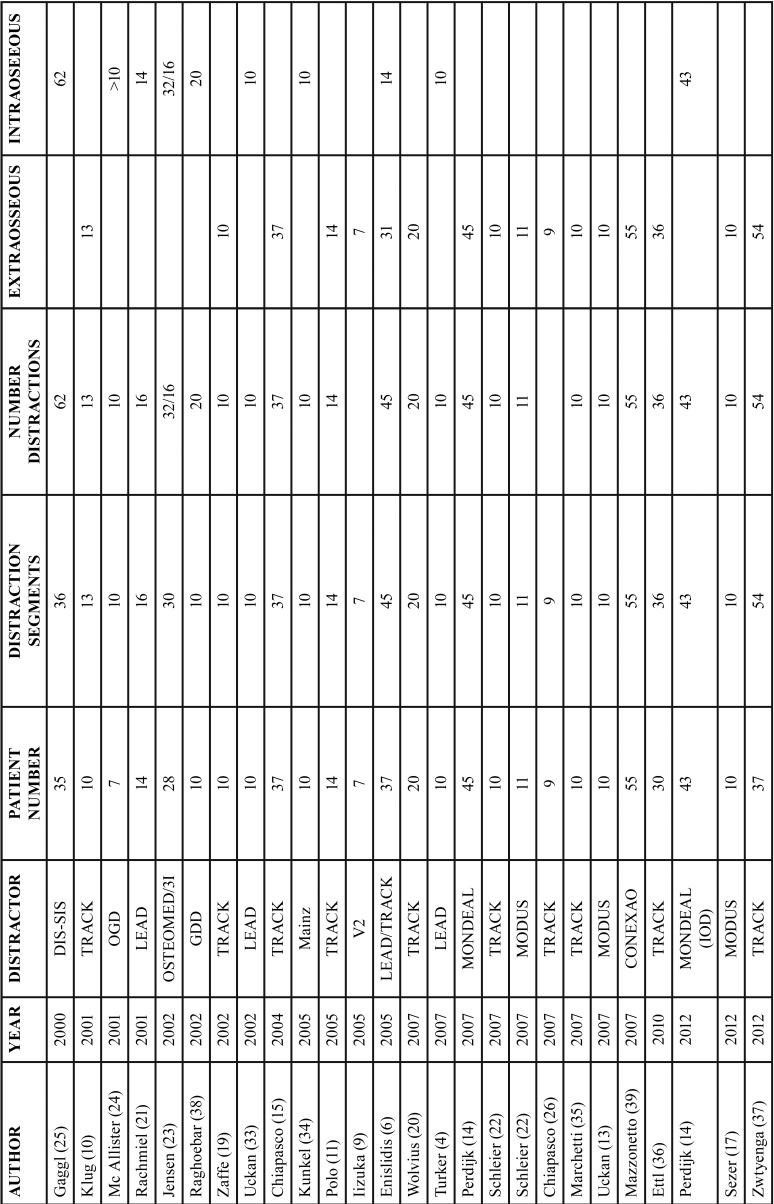
Type of distractor and number of patients.

**Table 2:**
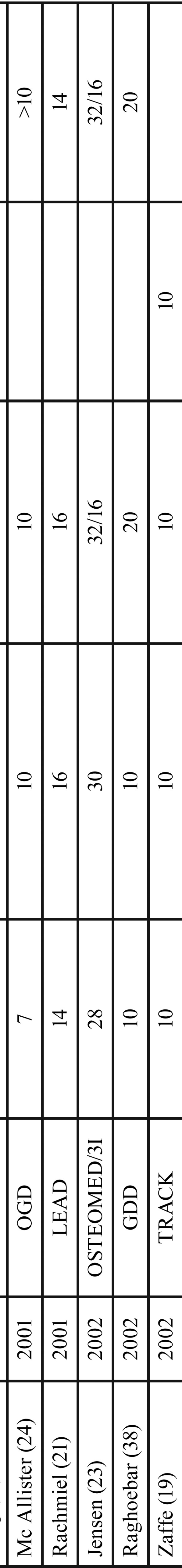
Type of distractor and number of patients.

In 13 studies the TRACK extraosseous distractor was used to treat a total of 231 patients, meaning this was the most common distraction device. The MODUS extraosseous distractor and the LEAD intraosseous distractor, were used in 5 studies each, treating 50 and 65 patients, respectively. The rest of the distraction devices were used in just 1 study. The CONEXAO distractor was used just one time but had a higher relevance due to the sample size (55 patients).

The main gain achieved with this distraction technique was 7.55mm. The average gain for extraosseous distractors was 8.13mm and 6.97mm in the case of intraosseous distractors.

The most common extraosseous distractor was TRACK, achieving a mean gain of 8.38mm. Using this distractor, the study that achieved the highest gain was conducted by Zaffe *et al.*, with a 12 mm gain (19), whereas the one achieving the smallest gain was developed by Wolvius *et al.* with 4.95mm (20). LEAD was the most common intraosseous distractor, which was used in 5 studies with a mean gain of 8.51mm. This model achieved the highest mean gain. The study that achieved the highest distraction was developed by Rachmiel *et al.* with 10.3 mm (21), whereas the one with the least gain was developed by Pérez-Sayans *et al.* with a mean gain of 5.74 mm (16) ( Table 3).

**Table 3:**
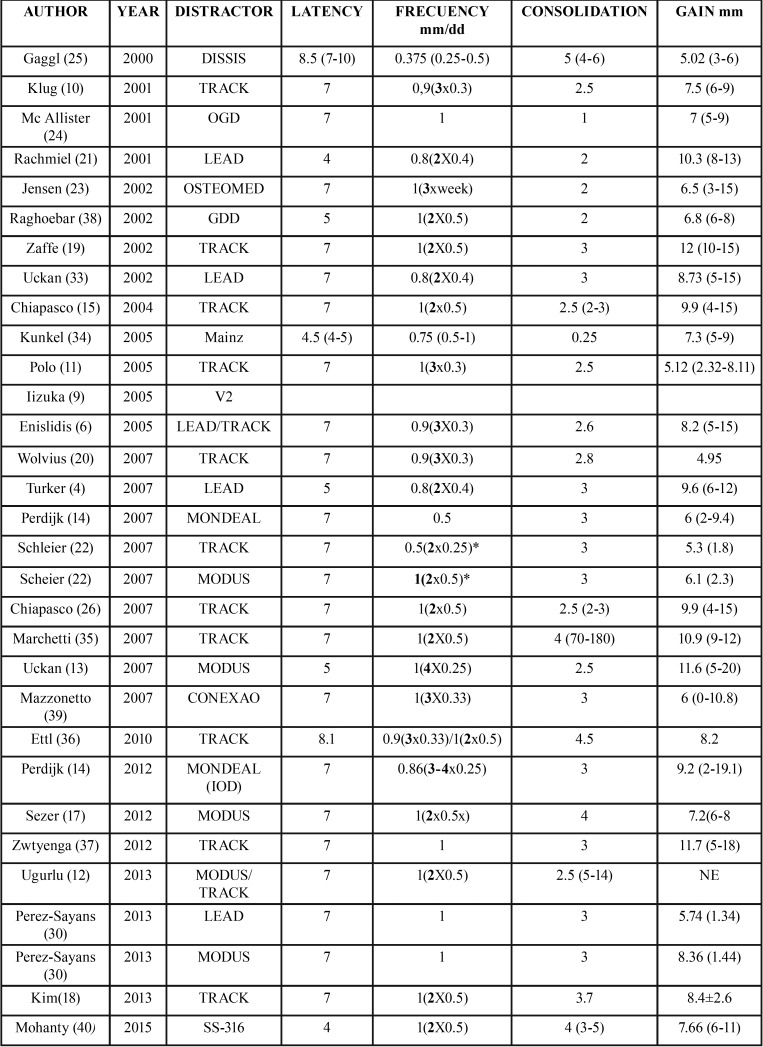
Distraction protocol used in different studies. * Mean distraction developed per day. In this protocol they are developed in increases (2x0.25mm the first 2 days and 2x0.5 the following days).

-Indication and location for distraction

The most frequent reason for distraction osteogenesis was bone atrophy (tooth loss, periodontal disease, prolonged use of removable prosthesis) amounting to a total of 442 cases, followed by problems caused by trauma (79 cases), tumor after-effects that required resection (42 cases). Cases of oligodontia, anodontia, sunken palate and osteomyelitis have also been described.

The most frequent location for the DO technique was the posterior area of the jaw (231), followed by the anterior section of the jaw or the interforaminal area (202), anterior maxilla or premaxilla (171) and posterior maxilla (30).

The ADOs developed on the maxilla and the mandible were 201 and 433, respectively, in other words over double the interventions in the lower jaw than in the upper jaw.

-Alveolar distraction osteogenesis protocol 

Most of the studies used a latency period of 7 days, with an average of 6.55 (4-10) days. The distraction period is the phase in which we found more discrepancies, since there are multiple sequences in terms of rhythm and frequency. All the authors agree in not going over 1 mm per day in terms of the distraction frequency. The mean distraction frequency was 0.88mm (0.375-1mm) per day.

In regards to the distraction rhythm, there is also a broad variation, from 1 to 4 times per day, spreading the daily frequency in equal periods. Some studies even develop the sequence in increases, starting with a distraction of 0.5 mm with 2 activations per day on the first 2 days, and increasing to 1 mm per day with 2 activations, the following days (22). Jensen *et al.* left 1 day to rest between activations, resulting in 3 activations of 1 mm each per week (23). The mean activation rhythm was 2.14 (1-4) times per day. In 7 studies they developed 1 activation per day, in 11 studies they developed 2 activations per day, in 7 other studies they developed 3 activations per day and in 4 studies they reached 4 activations per day.

In most of the studies, the consolidation period ranged between 2 and 4 months. There are certain exceptions, like the study by Mc Allister *et al.* in which the consolidation period only lasted 1 month (24); and Gaggl *et al.* who waited up to 4-6 months (25). The average consolidation period of the reviewed studies was 2.87 (1-5) months.  Table 3 shows in detail the latency, distraction, and consolidation periods of the reviewed studies.

-Complications and Treatment

In the 618 patients who underwent the ADO technique, there were a total of 507 complications, of which 430 (84.81%) were minor complications and 76 (15.18%) were major complications By order of frequency, the minor complications were the following: bad inclination of distraction vector: 133 (26.33%); insufficient bone breadth: 88 (13.36%); dehiscences: 60 (11.83%); paresthesias: 43 (8.48%); soft tissue problems: 49 (9.66%); pain: 21 (4.14%); infection: 20 (3.94%); insufficient height: 11 (2.17%).

Organized using the same criteria, the major complications were: mechanical problems: 29 (5.71 %); fracture of the basal bone: 19 (3.75%); fracture of the distractor: 17 (3.35%); fracture of the transport segment: 9 (1.77%); hypoesthesia: 5 (0.98%); loss of basal anchoring: 3 (0.59%).

In our review, we found a total of 117 additional grafts developed prior or during the implant surgery. They also developed 29 vestibuloplasties, to improve the conditions of the soft tissues and 8 osteotomies to regularize hard tissues.

Implants, loading time, follow up, success and survival.

The total number of implants placed after the ADO amounted to at least 1,280 implants, since not all the studies include these variables. The survival rate was 96% and the success rate was 95%, with a mean follow-up period of 32 months. The mean loading time after the implant placement was 4 months ( Table 4).

**Table 4:**
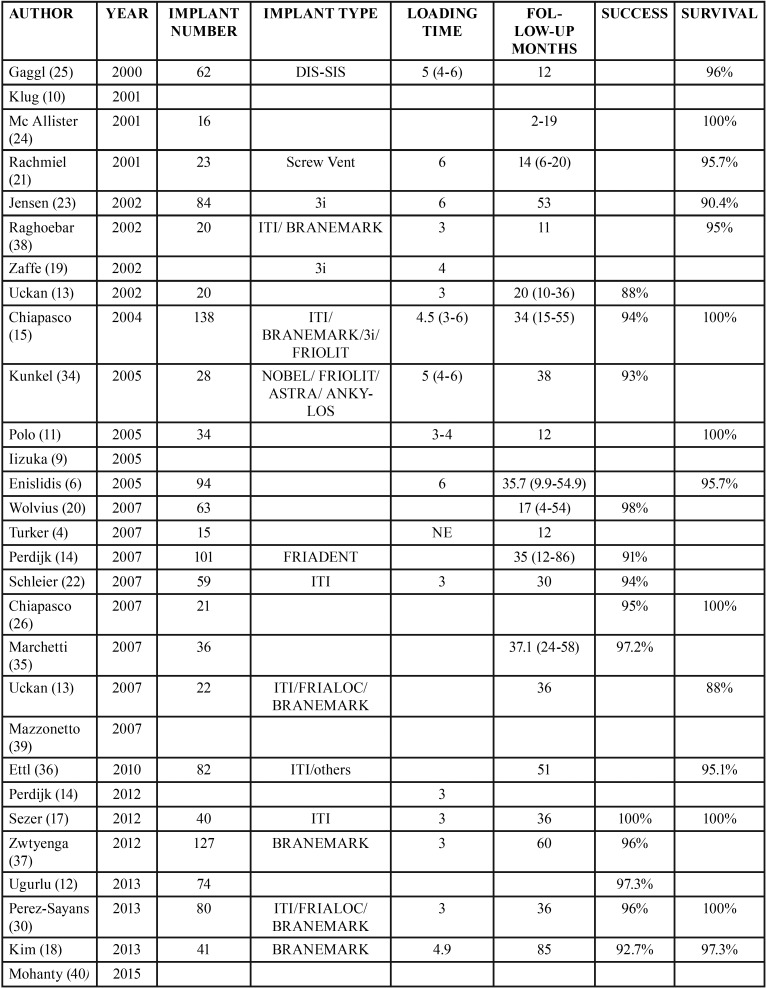
Implants placed in distracted bone, load time and follow-up.

The study in which most implants were placed was developed by Chiapasco *et al.*, with 138 implants (15). The greater follow-up period, after implant placement, was found in the study by Kim *et al.* who developed a follow-up of 85 months, observing a success rate of 92.7% and a survival rate of 97.3% within this time period (18).

Several authors achieved a survival rate of 100% (11,15,17,21,26), although no authors obtained this percentage in terms of the success of these implants.

## Discussion

A total of 29 articles were selected in this review about ADO. After over 20 years of use, there is still controversy regarding the best protocol to develop ADO. Different devices and applications have been developed, which have been used for different distraction protocols. However, the clinical phases of the distraction technique remain the same: Osteotomy, latency, distraction and consolidation.

The distraction period is the time ranging between the initial activation and the final activation of the distractor. Within the distraction period, we distinguish two different concepts: the distraction frequency (amount of bone distracted daily or the daily distance that is gained between the transport fragment and the basal bone. Measured in length, usually in millimeters.) and the distraction rhythm (the number of activations per day). There are multiple sequences in terms of rhythm and frequency. All the authors agree in not going over 1 mm per day in terms of the distraction frequency. Having found frequencies ranging between 0.375 and 1mm. Previous reviews found a daily mean distraction of 0.71 ± 0.27 and 0.85mm per day (27). We found a mean distraction of 0.88mm per day, from 2007 almost all the authors have used 1mm per day, which is why we’ve found a discrepancy with the mean calculated by Saulacic *et al.*

In regards to the distraction rhythm, there is a great variability, ranging from 1 to 4 times per day, spreading the daily frequency in equal parts. Some studies even develop the sequence in increases, starting with a distraction of 0.5 mm with 2 activations per day on the first 2 days, and increasing to 1 mm per day with 2 activations, the following days (22). Jensen *et al.* allow for 1 resting day between activations, developing 3 activations of 1 mm per week (23). This phase is very delicate, and the activations must be developed by especially trained staff. Therefore, a distraction rhythm of more than 2 times per day would complicate the chances of it being developed by the appropriate staff.

The mean consolidation of the revised studies was 12.05 (4-20) weeks, similar to the findings of Saulacic *et al.* and Rodriguez-Grandejean *et al.* who registered 12.22±5.58 and 11.83 weeks, respectively. In this period, the maturing and corticalization of the neoformed bone takes place. According to Amir *et al.* a minimum of 10 weeks is needed for the formation of the new bone bridges in a 10mm distraction gap (5). In most studies, the consolidation period ranges between 2 to 4 months. There are certain exceptions, such as the study by Mc Allister *et al.* in which they wait just one month for consolidation (24). While, Gaggl *et al.* wait up to 4-6 months, however they use a distractor-implant, which develops a prosthetic load. We have not found later studies reporting on the performance of this implant-distractor (25).

The mean gain of the study obtained by ADO was 7.55mm. The gain was greater with extraosseous distraction devices in contrast with intraosseous ones, ranging from 8.13 to 6.97mm. Saulacic *et al.* found a mean gain of 6.88±2.52mm (27). All the reviews on the vertical increase techniques agree that ADO is the technique with greatest potential in terms of vertical gain (1,26).

There are several criteria for the classification of the complications deriving from the ADO technique; Saulacic *et al.* (27) classified them depending on the moment in which the complication takes place, however, we have used the classification used by Enislidis *et al.* (6) who classified the complications into major and minor, this way we achieve a better quantification, since not all studies specify when these complications occurred.

Most of the complications we found in these reviews were minor, 430 (84.81%) and only 76 (15.18%), were major complications. The resolution of these minor complications is simple and doesn’t hinder the final result of the technique (6,27). A conservative treatment is recommended for minor complications such as inflammations, infections, temporary paresthesias or dehiscences. If the dehiscence is very large, reducing the rhythm and distraction frequency is recommended. Poor inclination of the distraction vector is the most common problem we found in 133 cases (26.33%), which can be corrected by traction, through orthodontics or by osteotomy or ROG if it were necessary.

According to Saulacic *et al.*, despite the high amount of complications, only 3.44% of them compromised the ultimate placement of the implants (27).

In terms of the deficiency of the width and height, the complications are quite frequent, amounting to 13.36% and 2.17%, respectively. These are solved using the ROG technique or bone grafts, depending on the necessary volume, before or during implant surgery. In our review, we found a total of 117 additional grafts developed prior or during the implant surgery. They also developed 29 vestibuloplasties, to improve the conditions of the soft tissues and 8 osteotomies to regularize the hard tissues.

The total number of implants placed after the ADO was at least 1,280 implants, since not all the studies included these variables. Previous reviews accounted for a total of 469 (27) and 652 (28) implants. The survival rate is 96% and the success rate is 95% with a mean follow-up period of 32 months. Milinkovic and Cordaro revised 351 implants placed after the ADO, finding a survival and success rate of 98.8% and 92.3%, respectively (29). The mean loading time after implant placement was 4 months. The study in which most implants were placed was developed by Chiapasco *et al.*, with 138 implants (15). The longest follow-up after implant placement was developed by Kim *et al.*, who followed up during 85 months, observing a success rate of 92.7% and a survival rate of 97.3% during this period.

Several authors achieved a survival index of 100% (26,30) although none of them reach this percentage when we are speaking of implant success. The survival of implants placed on the bone that have not been submitted to regeneration techniques amount to approximately 95%, which is similar to the rate that we found in the implants placed after ADO. This indicates that once the complications that this technique may produce are overcome, the performance of the implants will be as good as the implants placed on the bone that has not been submitted to any increase technique.

Perez-Sayans *et al.* compared 50 implants placed in a group of patients who underwent ADO with 50 ADO-free patients. They analyzed the periimplant loss at the loading moment, 1 and 3 years later. They found significant differences at the loading time, with a greater resorption in the ADO group (0.5mm in the ADO group and 0.25mm in the control group), after one-year follow-up period, there was no difference in the periimplant bone loss in both groups (0.66mm). But at the 3-year follow-up, the bone loss was significantly higher in the ADO group (1.03mm Vs 0.68mm) (30). The influence of prior bone defects that the ADO group had on these results stands out, amounting to 51% and 58% of cases, according to Saulacic *et al.* (27,31) since additional regeneration techniques were necessary. Periimplant bone loss was significantly higher in implants placed on bones that were subject to alveolar distraction in studies with a 3-4 year follow-up. However, these differences will continue to comply with the success criteria published by Albrektsonn (32).

Chiapasco *et al.* analyzed the performance in 138 implants following ADO four years later. They found a loss of 0.8mm in the first year after the functional loading. The following years the resorption was established at 0.1mm, for the second and third year, and 0.2mm on the fourth. They obtained a survival rate of 100% of the implants and a success rate of 94.2% (15).

Polo *et al.* found a resorption of 1.9mm one year after the load, in a total of 34 implants placed after the ADO in the posterior jaw area (11). In a later study, Chiapasco *et al.* compared the performance of 19 implants placed after the ADO and 21 after developing the graft with an autogenous block. We found similar results in both techniques, both in terms of success and survival rates as in terms of surgical and post-surgical complications (11).

## Conclusions

ADO is a technique with greater potential in vertical gain. The performance of dental implants has a success and survival rate similar to dental implants placed on bones that are not subject to increase techniques. The complexity of the technique, due to the number of variables and the numerous complications that take place during the ADO process are the greatest hurdle for the popularization of this technique. Further studies are necessary to assess whether alveolar distraction osteogenesis a useful technique.
